# Helminths and malaria co-infections are associated with elevated serum IgE

**DOI:** 10.1186/1756-3305-7-240

**Published:** 2014-05-23

**Authors:** Andargachew Mulu, Afework Kassu, Mengistu Legesse, Berhanu Erko, Demise Nigussie, Techalew Shimelis, Yeshambel Belyhun, Beyene Moges, Fusao Ota, Daniel Elias

**Affiliations:** 1 Department of Microbiology, College of Medicine, University of Gondar, Gondar, Ethiopia; 2 Aklilu Lemma Institute of Pathobiology, Addis Ababa University, Addis Ababa, Ethiopia; 3 School of Medical Laboratory Technology, College of Health Sciences, Hawasa University, Hawasa, Ethiopia; 4 Department of Preventive Environment and Nutrition, Graduate School of Nutrition and Bioscience, Institute of Health Biosciences, The University of Tokushima, Tokushima 770-8503, Japan; 5 ACE Biosciences, Unsbjergvej 2A, 5220 Odense, SOE, Denmark; 6 Institute of Virology, Faculty of Medicine, Leipzig University, Leipzig, Germany

**Keywords:** Helminths, Malaria, Th2, IgE, Ethiopia

## Abstract

**Background:**

Both helminth and malaria infections result in a highly polarized immune response characterized by IgE production. This study aimed to investigate the total serum IgE profile *in vivo* as a measure of Th2 immune response in malaria patients with and without helminth co-infection.

**Methods:**

A cross sectional observational study composed of microscopically confirmed malaria positive (N = 197) and malaria negative (N = 216) apparently healthy controls with and without helminth infection was conducted at Wondo Genet Health Center, Southern Ethiopia. A pre-designed structured format was utilized to collect socio-demographic and clinical data of the subjects. Detection and quantification of helminths, malaria parasites and determination of serum IgE levels were carried out following standard procedures.

**Results:**

Irrespective of helminth infection, individuals infected by malaria showed significantly high levels of serum IgE compared with malaria free apparently healthy controls (with and without helminth infections). Moreover, malaria patients co-infected with intestinal helminths showed high level of serum IgE compared with those malaria patients without intestinal helminths (2198 IU/ml versus 1668 IU/ml). A strong statistically significant association was observed between malaria parasite density and elevated serum IgE levels (2047 IU/ml versus 1778 IU/ml; P = 0.001) with high and low parasitaemia (parasite density >50,000 parasite/μl of blood), respectively. Likewise, helminth egg loads were significantly associated with elevated serum IgE levels (P = 0.003).

**Conclusions:**

The elevated serum IgE response in malaria patients irrespective of helminth infection and its correlation with malaria parasite density and helminth egg intensity support that malaria infection is also a strong driver of IgE production as compared to helminths.

## Background

Malaria remains one of the major causes of morbidity and mortality in the developing world and about 70% of the cases and 90% deaths are from Africa [1,2]. Similarly, helminthic infections are widely distributed in tropical and sub tropical areas of the developing countries. Helminthic infections are estimated to cause one million deaths per year and affect the health status of an individual on its physical, mental development and cause malnutrition, anaemia, stunted growth, cognitive impairment and lowered educational achievement [3]. Concurrent infection with helminths has been recognized as a possible confounding factor modulating the immune response to other pathogens [4,5]. Chronic helminthic infection results in a highly polarized immune response characterized by elevated T-helper cell type 2 cytokine, IgE production, eosinophilia [6] and are associated with increased immunological reactivity [5-8].

In sub Saharan Africa, due to the high prevalence of helminthic infections, a dominant Th2 immune response has been suggested to increase susceptibility to malaria, *M. tuberculosis* and HIV [9] and to hasten progression of these diseases [6,10,11]. Such an imbalance with an increase in Th2 cells favors IgE production [12], which may influence the clinical features of the disease. The immunological reports on interactions between malaria and helminths are still controversial. For example, the observation of high anti-*P. falciparum* IgE levels with a reduced risk of developing clinical malaria suggests the involvement of IgE in protection [13,14]. In contrast, the observation that circulating levels of IgE often correlate with severe rather than uncomplicated malaria suggests a pathogenic role of IgE [15,16]. A recent study from malaria endemic areas of Gabon and India showed that circulating levels of total IgE do not appear to correlate with protection or pathology of malaria [17].

In Ethiopia, malaria has been consistently reported as one of the three leading causes of morbidity and mortality in the past years, although a declining trend has been observed in recent years [18]. Like other developing countries Ethiopia is also endemic for helminthic infections [19-24]. We and others have reported malaria-helminth co-infecton rates and the possible impact of helminthes infection on prevalence and clinical outcomes of malaria [24-26] and the impact of deworming [25,27,28]. However, data on the relationship of the host immune response correlates during malaria-helminths co-infection are lacking. Thus understanding the immune response during malaria-helminth co-infection will maximize the probability of identifying new targets for the design and development of immunotherapeutic approaches and the prevention and control of both infections in highly endemic areas. This study was conducted to investigate the IgE profile *in vivo*, as a measure of the Th2 immune response, among malaria patients with and without helminth co-infection from south Ethiopia where the epidemiological coexistence of these infections is very high.

## Methods

This cross sectional observational study was conducted at Wondo Genet Health Center, Southern Ethiopia in 2006/2007. The inclusion criteria of the study subjects and the settings were briefly described earlier [25]. However, in this study adult patients who were microscopically positive for malaria (N = 197) with an age range of 20–49 years old were only included. Apparently healthy individuals upon physical examination, free from signs and symptoms of acute febrile illness for the last two weeks from the same endemic locale (N = 169) and from malaria non-endemic Ethiopian highlands (N = 47) were also used as controls. All the healthy controls were microscopically negative for *Plasmodium* species and all the subjects were naïve for anthelminthic or anti-malarial drugs for four weeks time prior to data collection.

A pre-designed structured format was used to collect socio-demographic and all relevant clinical data of the patients. After getting written and/or verbal informed consent, 5 ml of venous blood was collected in vacutainer tubes. Then when the clot had retracted serum was separated and stored at −20°C until used for measurement of serum.

Both thick and thin blood films were made in a single slide and were stained with Giemsa’s staining solution for detection and quantification of malaria parasites [MOH, Standard Malaria Diagnosis and Treatment Guideline, 2004]. To detect malaria infections, 200 fields (the equivalent of 0.5 μl of thick blood film) were examined as described before [25]. Briefly, the parasite density was expressed per micro liter [μl] of blood assuming 8000 leucocytes per μl of blood. In brief, a thick film was selected where the white blood cells were evenly distributed. Using the oil immersion objective, 200 white blood cells were counted systematically, by counting at the same time the number of parasites (asexual form only) in each field was covered. Then, the number of parasite per μl of blood was calculated by multiplying the number of parasite (asexual stages) counted against 200 leucocytes and 8000 leucocytes and dividing the product by 200 [29].

The presence of intestinal parasites was detected from stool samples using direct microscopy and formol-ether sedimentation techniques. Moreover, coarse quantification of eggs was obtained by counting the number of eggs on a smear of 41.7 mg of feces, and a quantitative variable scoring (light infection/low worm burden, moderate infection/medium worm burden and heavy infection/massive worm burden) was created for each helminth following the standard procedure used by WHO [29,30].

The total serum IgE levels were quantified by total IgE ELISA kit (IBL Immunobiological Laboratories, Hamburg, Germany) following the manufacturer’s instructions as described earlier [25]. Briefly, 10 ml serum samples or standard IgE were pipetted in duplicate into wells of microtiter plates precoated with monoclonal mouse antihuman IgE antibody together with per-oxidase conjugated antihuman IgE. After incubation for 30 min at room temperature the plates were rinsed with diluted wash buffer to remove unbound material. Then a substrate solution (tetra methyl benzidine) was pipetted and incubated for 15 minutes to induce development of color. The reaction was terminated by the addition of stop solution and the resulting dye was measured in a spectrophotometer at a wave length of 450 nm against the substrate blank. The IgE concentration of the samples was read from a standard curve. Mean values of two separate determinations from each sample were used as serum IgE levels of a particular study subject. This was only done on 89 randomly selected malaria patients (20–49 years) with (n = 28) and without (n = 28) helminth co-infection; and malaria negative apparently healthy controls with (n = 20) and without (n = 20) helminth co-infection from malaria endemic areas and without helminth (n = 9) from malaria non endemic areas. Subjects found positive for intestinal protozoa (*Entamoeba histolytica* and *Giardia lamblia*) were not included for IgE determination. This is because of different IgE responses observed in protozoal and helminths infections.

The data was analyzed using to SPSS version 15 statistical package software. Intestinal parasite densities were transformed to log for analysis and geometric mean was used. Non-parametric tests were performed to compare mean values of the different groups. The Mann Whitney test and the Kruskall Wallis tests were used for comparisons between two groups and three or more groups, respectively. Spearman’s correlation was used to check for correlations between parameters. P values were considered significant when found <0.05.

The study protocol and design including the consent procedures were approved by The Ethical Review Committee of the Aklilu Lemma Institute of Pathobiology, Addis Ababa University and South Nations and Nationalities Peoples Region Health Department. Written (from those who can read and write) or verbal (from those who can’t read and write) informed consent from all study subjects was also obtained. The written informed consent and the ethical clearance letters are documented in both offices. However, verbal consent was not recorded and documented. The consent procedures were also approved by both The Ethical Review Committee of the Aklilu Lemma Institute of Pathobiology, Addis Ababa University and South Nations and Nationalities Peoples Region Health Department. Patients with malaria and helminthiasis were managed as part of the routine clinical management of patients in the health care facilities following the national guideline [MOH, Standard Treatment Guidelines, 2004].

## Results

In this cross sectional observational study 197 malaria patients and 216 malaria free apparently healthy controls were included. The mean age of the study subjects in both groups was not different (34 versus 35 years). The male female ration was also 1: 1.2. The demographic characteristics of the pateints, magnitude of malaria and type and destitution of helminthes were described previously [25]. Fifty six percent of the malaria infections were due *P. falciparum. Plasmodium vivax* accounted for 35.2% of malaria cases. Mixed infections with *P. falciparum* and *P. vivax* were detected in 6% of the subjects. Irrespective of malaria infection, high rates of helminth infection were observed in the settings. There was no significant difference in magnitude of helminths among malaria patients and malaria free apparently healthy controls (78.2% (154/197) versus 84.3% (182/216)), respectively. The pattern of helminth infection in malaria positive and malaria free apparently healthy controls was similar (Table 1). The most frequent intestinal helminths detected were *Trichuris trichiura, Ascaris lumbricoides, Schistosoma mansoni* and hookworm. A small proportion of the patients were also infected with intestinal protozoan cysts of *Giardia lamblia* (2 patients) and *Entamoeba histolytica/dispar* (5 patients). The least encountered helminths were *Taenia* species (7), and *Strongyloides stercoralis* (2). Twenty-three percent of the subjects from both groups had a single infection. Multiple infection i.e. infection with 2, 3 and 4 different species of intestinal parasites were observed in 37.0% (132/356), 21.6% (77/356) and 3.4% (12/356) patients, respectively. The frequent combinations of helminths diagnosed in single patients were *A. lumbricoides - T. trichiura* in 28.8%; *A. lumbricoides - T. trichiura - S. mansoni* in 18.5%, and *A. lumbricoides -T. trichiura*- Hookworm in 8% of the patients. In 4% of the patients 4 different helminths: *A. lumbricoides, T. trichiura, S. mansoni* and hookworm were observed.

**Table 1:** **Magnitude of intestinal helminths**^**a **^**in malaria patients and malaria free apparently healthy controls (numbers given in brackets percentages)**

**Type of helminths**	**Malaria**	**No malaria**	**Total**	**OR**	***P*** **†**
	**(N = 197)**	**(N = 216)**	**(N = 413)**	**(95%CI)#**	
*T. trichiura*	119 (63.6)	98 (58.0)	217 (61.0)	1.361 (1.119, 1.655)	0.002
*A. lumbricoides*	98 (52.4)	98 (58.0)	196 (55.1)	1.147 (0.780, 1.687)	1.147
*S. mansoni*	52 (27.8)	43 (25.4)	95 (26.7)	1.137 (0.976, 1.326)	1.137
Hookworm	23 (12.3)	19 (11.2)	42 (11.8)	1.086 (0.926, 1.275)	1.086

^a^:because of multiple infection the total is not summed up to 100; OR = Odds ratio; #CI = Confidence interval; † = P value.

**Figure 1:**
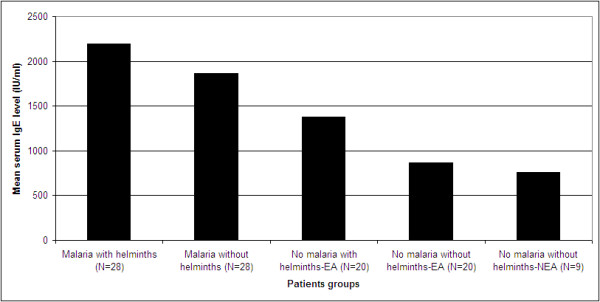
**Mean serum IgE levels in adult malaria patients with and without helminths infection and malaria free health controls without and with out helminths coinfections.** EA: malaria endemic area; NEA: malaria non-endemic area.

**Figure 2:**
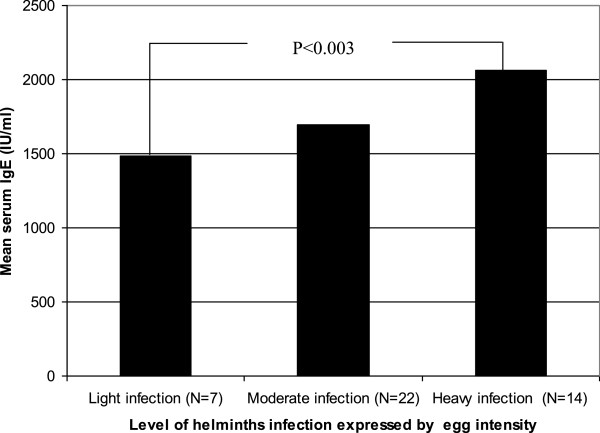
Correlation between helminthic load and serum IgE level helminths and malaria infected individuals.

Although, it is very difficult to have a normal range of IgE even at a population level, a range of values of IgE levels was defined enabling the analysis of the frequency of normal, moderate and high IgE levels in each group of patients. Normal values were adjusted because the groups had a wide range of different total serum IgE level. Thus, the normal value (N) was defined by the median IgE levels from malaria free apparently healthy helminth negative controls from malaria non endemic area. Accordingly, serum IgE values between N and 1.5 N were considered as low, between 1.5 N and 2 N as moderate IgE levels and above 2 N as the highest levels.As indicated in Figure 1, irrespective of helminth infection, individuals infected by malaria showed significantly high levels of serum IgE compared with malaria free apparently healthy controls (with and without helminth infections). Moreover, malaria patients co-infected with intestinal helminths showed high levels of serum IgE compared with those malaria patients without intestinal helminths (2198 IU/ml versus 1668 IU/ml). There was no statistically significant (P > 0.05) difference in mean serum concentrations of IgE between falciparum and vivax malaria (2165 IU/ml versus 1978 IU/ml; P = 0.063) irrespective of helminth co-infection. However, a strong statistically significant association was observed between malaria parasite density and elevated serum IgE level (2047 IU/ml versus 1778 IU/ml; P = 0.001) for high and low parasitaemia (parasite density >50,000 parasite/μl of blood), respectively. Likewise, significant association (P = 0.003) was found between helminth egg load and serum IgE levels in both malaria positive and malaria free apparently healthy controls with helminth infection after controlling for malaria infection (Figure 2). No significant association was found between presence of individual intestinal parasites and serum IgE level (P = 0.089). Individuals with helminth-helminth coinfection had slightly higher mean serum IgE levels than those infected with single parasites (1894 IU/ml vs. 1654 IU/ml). In both groups levels of serum IgE was not correlated with age and gender differences (P = 0.45).

## Discussion

The finding of elevated serum IgE level among malaria patients irrespective of helminth co-infection and the high serum IgE concentration in malaria free helminth infected apparently healthly controls is in agreement with previous studies [17,31,32] and supports the data that both malaria and helminth infection shifts the immune response to Th2 type. Interestingly, this study is the first in its kind to report the association between malaria parasite density and helminth egg intensity with elevated serum IgE level. The highly elevated serum IgE level in malaria infected patients with helminth co-infection in the current study might be a result of both malaria and helminth induced immune dysregulation which induces a shift in cytokine production from Th1 to Th2 [32] and the resulting polyclonal activation of B cells in which the production of cytokines (including IL-4 and IL-6) from Th2 cells increases synthesis of IgE. Further more the higher IgE levels in malaria free apparently healthy controls without helminth infection from both malaria endemic and non endemic areas may be due to environmental factors and genetic background, which may predispose to the development of IgE [33]. Although, a significant increase in IgE levels with age was reported from other malaria endemic African countries [33], which may be a reflection of an increase in the capacity of the immune system to respond to parasite infections, we do not observe such correlation. This would be expected because the study groups in the current study comprised exclusively adults ranging from 20–49 years old.

The correlation of malaria parasite density and helminth egg intensity with elevated serum IgE levels could also contribute to severity of disease. As it was reported [15,16], total IgE elevation has been described as a pathogenic factor during malaria. In support of this we have also observed significantly high serum IgE levels with parasite density >50,000 parasite/μl of blood. However, serum IgE level was not different in different clinical forms of malaria (uncomplicated, severe and cerebral malaria) unlike previous report from Gabon and India [17]. This could be the hyper endemicity of malaria in the present study group which suggests that exposure to the parasite strongly influences the production of IgE, although this difference may also be due to other factors as well. Nevertheless, it was reported that IgE levels were higher in patients with severe malaria infection. Although, total IgE level increased in helminth infection, it usually does not eliminate worms [32]. Moreover, evolution seemed to have selective antigenic determinants with potent IgE-inducing capabilities. For example *Ascaris* is highly allergenic; if the function of IgE was only to get rid of worms; it would seem detrimental for *Ascaris* to induce it. However, no significant association was found between presence of individual helminths and serum IgE levels in the current study. The prevalence of intestinal parasites among malaria patients and malaria free apparently health individuals were as high, which corresponds well with previous studies from Ethiopia [22-25] and shows the endemicity of helminthiasis in the area. We and others have previously reported elevated IgE levels from untreated TB-HIV co-infected patients with and without helminth co-infection indicating an *in vivo* evidence for Th2 activation [32,34-36]. Thus the findings of the current study are also additional evidence for the change in the immune system during malaria and heleminth concurrent infection towards T helper −2 dominant types.

## Conclusions

Despite the low sample size and the cross-sectional nature of the study, a remarkably elevated total serum IgE level in malaria patients irrespective of helminth co-infection was observed. The elevated serum IgE response in malaria patients irrespective of helminth infection and its correlation with malaria parasite density and helminth egg intensity support that malaria infection is a strong driver of IgE production as helminth. Thus, mass deworming may be a cost effective approach in reducing the morbidity of helminths and co-infection where malaria, tuberculosis and HIV are endemic.

## Competing interests

The authors declare that they have no competing interests.

## Authors’ contributions

AM: conception of the research idea, study design, data collection, analysis and interpretation and the drafting of the manuscript; YB and BM: reviewing the manuscript; ML and BE: designing the study and reviewing the manuscript; DN: collecting data, reviewing the manuscript; TN, FO, AK and DE: designing the study, reviewing the manuscript. All authors read and approved the final version of the manuscript.
